# Cognitive Behavioral Immersion for Depression: Protocol for a Three-Arm Randomized Controlled Trial of Peer-Based Coaching in the Metaverse

**DOI:** 10.2196/65970

**Published:** 2025-05-06

**Authors:** Noah Robinson, Francisco N Ramos, Steven D Hollon, Gloria T Han, Iony D Ezawa

**Affiliations:** 1 Department of Psychology Vanderbilt University Nashville, TN United States; 2 Department of Psychology University of Southern California Los Angeles, CA United States

**Keywords:** cognitive behavioral immersion, virtual reality, anxiety, mental health, depression, stress, protocol, randomized controlled trial, RCT, coaching, metaverse, behavioral therapy, skills training intervention

## Abstract

**Background:**

Depression and anxiety are among the most common mental health concerns globally. Efficacious treatments such as cognitive behavioral therapy exist but remain difficult to access and scale. Cognitive behavioral immersion (CBI)—a cognitive behavioral skills training intervention delivered by peer coaches in the metaverse—has been developed to address these barriers. CBI can be used through a virtual reality headset or via flat-screen devices such as phones, tablets, or computers. Pilot data have established its usability among participants with clinical levels of depression and anxiety. However, more research is needed to determine whether CBI causes decreases in these symptoms and how delivery via virtual reality compares to flat-screen devices.

**Objective:**

This protocol aims to conduct a randomized controlled trial evaluating the efficacy of immersive CBI accessed via a virtual reality headset (CBI-VR) as compared to a less immersive (but more accessible) CBI condition accessed via a flat-screen device (CBI-FS) and each to a delayed access control (DAC).

**Methods:**

A total of 306 adults experiencing clinical levels of depressive symptoms are being recruited nationally to participate in this web-based trial. Participants will be randomized according to a 1:1:1 ratio to one of three conditions: (1) CBI-VR, (2) CBI-FS, and (3) DAC. The CBI program consists of eight weekly 60-minute group sessions led by trained peer coaches who teach cognitive behavioral skills. The acute period of each condition will last 8 weeks with a follow-up period of 6 months. The primary outcome is depressive symptoms; secondary outcomes are anxiety symptoms and quality of life. Outcomes will be assessed once at baseline, weekly during the course of the intervention, and monthly during follow-ups. We will use hierarchical linear models to assess differences in the rate of symptom change among conditions. We will also explore potential prognostic (demographics and immersion) and prescriptive (cognitive behavioral skills, group alliance, and program engagement) predictors, as well as potential mechanisms (cognitive change and social support) of response.

**Results:**

We hypothesize that participants randomized to either CBI group will experience greater symptom improvement than those in DAC and that CBI-VR participants will improve more than CBI-FS participants. This study was funded in September 2023. Data collection began in February 2024. As of January 2025, all 306 participants have been enrolled. Data collection should conclude by September 2025. Data have not yet been analyzed. Expected results to be submitted for publication in the winter of 2025.

**Conclusions:**

This trial will determine if CBI via either device is efficacious as compared to DAC and whether virtual reality enhances outcomes. Findings will contribute to the literature on using the metaverse and virtual reality to facilitate effective accessible mental health interventions, particularly for depression.

**Trial Registration:**

ClinicalTrials.gov NCT06418997; https://clinicaltrials.gov/study/NCT06418997

**International Registered Report Identifier (IRRID):**

DERR1-10.2196/65970

## Introduction

### Background

Approximately 280 million people worldwide are experiencing depression [1], and nearly half of those people also experience comorbid anxiety [2]. Symptoms of either disorder can cause substantial distress and impair functioning across all domains of life. Worse yet, the prevalence and severity of these disorders are growing [3]. Despite the increasing evidence base of effective interventions, including cognitive behavioral therapy (CBT), the prevalence of depression and other mental health disorders continues to rise [4]. Accordingly, there is a demand for effective, accessible, and scalable interventions to manage these mental health conditions.

CBT is one of the most effective interventions for depression and anxiety [5]. The model underlying this intervention describes how one’s cognitions influence and are influenced by their emotional, behavioral, and even physiological consequences [6]. Based on this model, CBT teaches clients a variety of cognitive behavioral skills to identify, evaluate, and change maladaptive thoughts and behavioral patterns to indirectly change emotions. CBT has been deemed an empirically supported treatment through direct comparisons to waitlist controls and other bona fide interventions such as antidepressant medications [7]. Not only is CBT generally effective during its acute phase but it also has an enduring effect on symptoms that lasts beyond the end of treatment [8]. Despite its many strengths, CBT-oriented care is often difficult to access and scale due to the lack of trained providers, high costs, and stigma associated with seeking mental health care [9].

Two major innovations in the mental health care field could help to address the aforementioned problems of accessibility—the deployment of lay therapists and the use of technology. First, nonprofessional lay therapists (hereafter referred to as “coaches”) can be trained to provide peer support and teach cognitive behavioral skills. A primary driver of the effects seen in CBT is clients’ use of the specific cognitive behavioral skills taught [10,11], suggesting that cognitive behavioral skills may be a worthwhile training target for mental health support. Prior research has shown that individuals without any prior formal professional training can be taught to deliver effective cognitive behavioral interventions [12], therefore, reducing the burden on and expense of the limited workforce of highly trained, specialized therapists.

Second, digital mental health interventions can deliver cognitive behavioral skills training nearly anywhere at any time and have outcomes comparable to face-to-face CBT [13-18]. Although technology can remove the need for therapists (eg, through completely self-guided interventions or provided through the use of artificial intelligence–based computerized cognitive behavioral programs), digital CBT programs have experienced high dropout rates, with reviews reporting average dropout rates of 32%-74% depending on dropout definition and level of support [19-21]. Fortunately, human support provided via peers or coaches can help improve accountability, engagement, and efficacy [22]. Furthermore, human-mediated digital mental health interventions can help promote nonspecific effects that may contribute to outcomes [23]. Social connection has been posited as a key contributor to engagement in digital mental health interventions [24], indicating that digital interventions must strike a balance between accessibility and human interaction in order to be scalable while remaining effective. While existing CBT programs have aimed to leverage lay therapists and digital modalities to deliver interventions remotely, at scale, and at a lower cost, their uptake and effectiveness remain inconsistent. This highlights the clear need for innovative approaches that can enhance both engagement and efficacy.

Recently, the technology of the metaverse, defined as internet-connected social digital environments that allow end users to interact as avatars, has been adopted to facilitate digital mental health interventions delivered by humans [25]. The metaverse can transport an individual into a personalized 3D digital world that promotes immersion, the extent to which an individual perceives, experiences, and interacts with a digital environment as if it were a real environment [26]. Individuals can create an anonymous avatar and become immersed in a world where they communicate by speaking to other people, each of whom is also represented by an avatar. This technology affords a unique opportunity to deliver an intervention that is simultaneously immersive and anonymous, potentially allowing individuals to feel more comfortable being vulnerable [25]. The level of anonymity that accompanies the use of avatars may also attract end users who typically do not seek out mental health care, whether due to shyness or stigma [25,27]. Finally, the digital nature of the metaverse can reduce barriers to access related to cost, lack of trained providers, or other physical hurdles. Metaverse environments can be accessed through commonly owned flat-screen devices (eg, computer or phone) or through virtual reality technology (ie, head-mounted displays that generate graphics in real time that simulate a 360° view of an environment that one can interact with using hand-held controllers). Virtual reality enables end users to feel strongly immersed in a 3D space, which can increase the sense of presence in a particular environment despite being physically situated in a different place [28]. The metaverse is a suitable setting for coach-based mental health interventions designed to facilitate cognitive behavioral skills training, integrating digital facilitation of effective mental health interventions with realistic anonymous interpersonal interactions. Despite growing interest in virtual reality and metaverse apps for mental health, research on integrating virtual reality and the metaverse with coach-supported CBT skills remains limited.

Cognitive behavioral immersion (CBI), a synchronous cognitive behavioral skills group training program delivered by coaches in the metaverse developed by Innerworld, Inc, is the first app to our knowledge that bridges virtual reality, the metaverse, and CBT skills in the pursuit of delivering efficacious, accessible, and scalable mental health care [25]. A pilot study provided support for its feasibility among a group of individuals in recovery from a substance use disorder [25]. A second study on this intervention demonstrated improvement in depression and anxiety symptoms associated with CBI engagement [29]. That study found that internet-based social support was associated with depressive symptom improvement, while the use of virtual reality (vs a flat-screen device) with the intervention was associated with greater anxiety symptom improvement. While this preliminary support for the use of CBI is promising, a direct comparison of this modality against a control has not yet been conducted, and its absolute efficacy is unknown. In addition, it is unknown whether accessing CBI via immersive virtual reality confers any advantage over more readily accessible, less immersive flat-screen devices.

### Objectives

This study is designed to address the following research questions: (1) is CBI efficacious (better than its absence)? and (2) does immersive virtual reality confer any advantage over and above accessing CBI via less immersive flat-screen devices? In line with these questions, this study aims to compare immersive CBI accessed via virtual reality (CBI-VR) to a delayed access control (DAC) condition and less immersive CBI accessed via flat-screen devices (CBI-FS) in a randomized controlled trial. We predict that participants randomized to CBI-VR will experience greater clinical and functional improvement than participants randomized to the other conditions. In addition to this primary aim, we plan to explore predictors of response regardless of condition (prognostic), prescriptive indices that predict differential program response (moderators), and causal mechanisms of clinical and functional change across conditions (mediators).

## Methods

### Study Design

**Figure 1:**
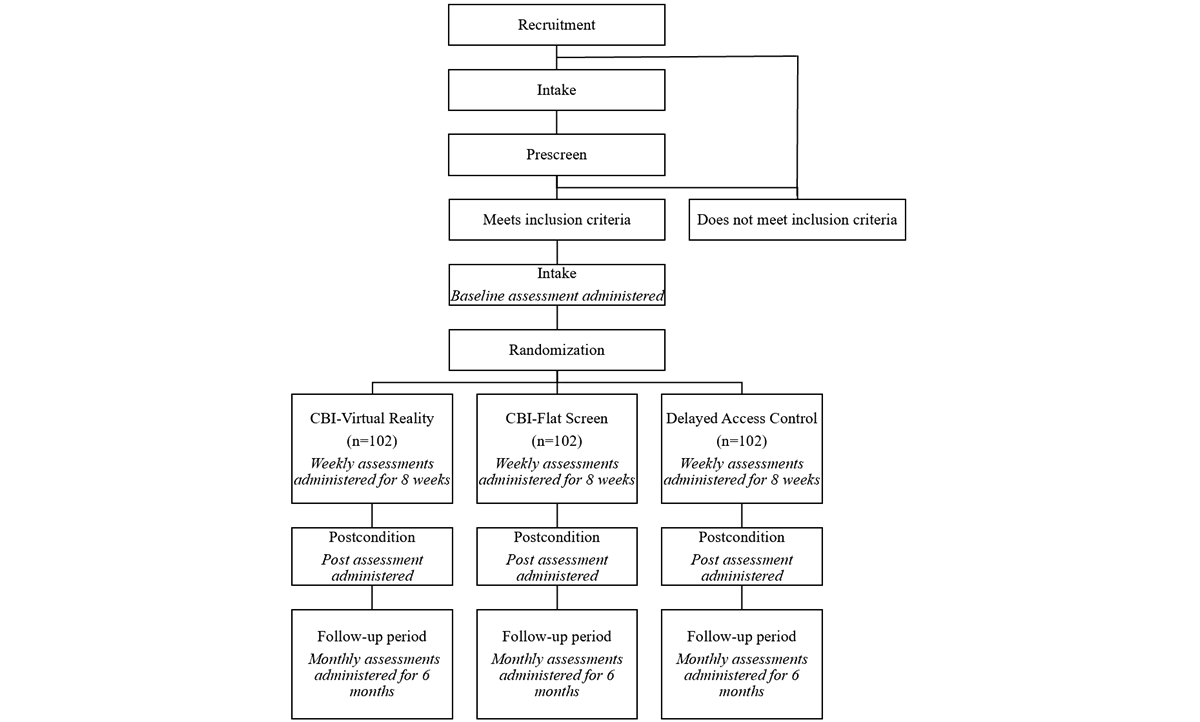
Flowchart of study procedures. CBI: cognitive behavioral immersion.

This study is a three-arm unblinded randomized controlled trial (NCT06418997). Participants are randomly assigned to one of three groups in a 1:1:1 ratio: CBI-VR, CBI-FS, or DAC. In the CBI-VR and CBI-FS conditions, participants participate in up to eight weekly intervention sessions. Those in the CBI-VR condition will be mailed Meta Quest 3 headsets to participate in the intervention, while those in the CBI-FS condition will use their own existing flat-screen device (such as a personal computer, tablet, or smartphone) to participate in the intervention. Participants in the DAC condition will be asked not to access the intervention for 8 weeks but will then be provided with information on and encouraged to access the same intervention. Outcomes will be monitored weekly throughout the 8-week acute phase and monthly for 6 months during follow-ups. All procedures will be conducted on web-based platforms. See Figure 1 for a flowchart of study procedures. Reporting in the protocol was based on the CONSORT-EHEALTH (Consolidated Standards of Reporting Trials of Electronic and Mobile Health Applications and Online Telehealth) and SPIRIT (Standard Protocol Items: Recommendations for Interventional Trials) checklists (Multimedia Appendices 1 and 2).

### Ethical Considerations

All study procedures received human participant ethics review approval from the University of Southern California’s (USC) Institutional Review Board (UP-23-00491). As part of the approved procedures, all participants will provide informed consent prior to beginning the study and be made aware that they can stop their participation in the study at any time. All data will be collected securely through REDCap (Research Electronic Data Capture; Vanderbilt University) [30,31]. After the study is completed, all data will be deidentified. Participants will be compensated with electronic gift cards sent to their email for completion of study surveys. Participants across all conditions will also receive a new Meta Quest 3 virtual reality headset to keep as part of study participation. Distribution of the headset is dependent on study randomization; those randomized to the CBI-VR condition receive a headset prior to starting their 8-week condition, whereas participants in the other two conditions receive the headset after completion of the 8-week acute condition.

### Participants

We are recruiting a sample of 306 individuals from across the United States who endorse clinical levels of depression symptoms. Participants are recruited through paid advertisements on popular social media apps (eg, Facebook and Instagram), posts on clinical trial or research study registries (eg, ClinicalTrials.gov), through our research lab’s website, and by word of mouth.

### Inclusion and Exclusion Criteria

The inclusion criteria for this study are (1) clinical level of depressive symptoms (indicated by a score of ≥10 on the 9-item Patient Health Questionnaire [PHQ-9] [32]), (2) 18 years or older, (3) able and willing to give informed consent, (4) personal computer with a stable internet connection that can run the CBI application, and (5) able to speak and read English. The exclusion criterion for this study is elevated suicide risk at the baseline assessment that precludes inclusion in the study (see Safety Monitoring section for more information).

### Prescreening and Intake Procedures

All prescreening and intake procedures will be managed by a research team at USC. Interested individuals will be directed to a link where they will complete an initial prescreening questionnaire hosted on REDCap containing a brief description of the study, eligibility questions, and the PHQ-9. Those who meet initial inclusion criteria will be emailed a link to schedule a 20-minute intake session conducted via Zoom (Zoom Video Communications).

Prior to attending their intake session, each individual will be asked to sign the study consent form via the REDCap eConsent module and to complete a set of intake measures via a secure REDCap link. During the intake session, study personnel will explain the study; answer any questions; confirm participant eligibility; record the participant’s name, address, and mobile phone number for verifying identity and for mailing the virtual reality headset; collect the participant’s availability for attending a CBI intervention group, and secure informed consent. After completing the intake session, participants will be randomized to one of three conditions.

Participants randomized to the CBI-VR or CBI-FS conditions will be sent an email alerting them of their study conditions and providing directions on how to access the application hosting the CBI intervention. Prior to their first intervention session, participants will take part in a web-based tutorial about how to navigate the CBI intervention and may attend an optional onboarding session with study personnel if additional support is needed navigating the CBI intervention (and virtual reality headset if randomized to the CBI-VR condition). DAC participants will also be emailed to inform them of their study condition, when they can expect to begin receiving surveys, and when they can expect to receive compensation.

### Randomization

Participants are randomized to one of the three groups in a 1:1:1 ratio using a random number generation Python script. The seed Python uses to perform random generation is predetermined for each “wave” of participants to aid replicability. Once the number of recruited participants in a wave has reached the cap indicated by coach and researcher availability, recruitment will be paused, and that wave of participants will be randomly assigned to a group and commence their 8-week condition. This process will repeat every few weeks until the planned sample size of 306 is reached. Randomization will be done in blocks so that participant distribution across each group per wave is equated to ensure CBI classes during each wave have sufficient scheduled participants. The randomization procedures are conducted by the research team at USC.

### Intervention

The CBI intervention will be hosted on the Innerworld app. Innerworld is an English language app freely available for download across several platforms (eg, Windows, Meta Quest headsets, iOS, and Android). Innerworld trains adults who do not necessarily have prior professional training to serve as CBI coaches. Each coach undergoes an intensive 20-hour training program spread over 5 days led by Innerworld support staff to learn how to facilitate cognitive behavioral skills groups. Coaches will be trained to lead an 8-week CBI for depression course, which was developed by investigators who are experts in CBT. See Table 1 for a condensed outline of the coach training program. After completion of the fifth training session, each coach must cofacilitate at least one real meeting in the presence of a trainer (ie, a coach who has extensive experience leading Innerworld groups). The coach is evaluated by the trainer and provided with feedback. To maintain coach adherence to the intervention throughout the trial, coaches will be required to attend weekly supervision led by Innerworld support staff and team investigators. We will also ask participants to complete the Group Session Rating Scale to assess group quality.

The CBI for depression intervention involves participants meeting in groups of up to 10 in which they learn lessons on various strategies to cope with depression based on cognitive behavioral theory [6]. Each group meets for 1 hour once a week for 8 weeks. In addition, user feedback during pilot testing led to the implementation of an “a la carte” model that allows participants to attend a rotating schedule of makeup sessions. Peer coaches will follow a detailed manual describing the content to be followed for each session. See Table 2 for a condensed outline of the session content. Behavioral strategies include but are not limited to activity scheduling, problem-solving, and assertion training. Each session begins with setting the agenda and reviewing homework from the previous session. Cognitive strategies include but are not limited to identifying the connections between thoughts, feelings, physiology, and behaviors, and catching and examining negative automatic thoughts. Each session ends with a session summary, homework assignment, and feedback about the session. If participants cannot attend their assigned session, participants are encouraged to attend a makeup for that session via email prompts sent by the research team. If participants need support navigating the app or using their virtual reality headset after beginning the intervention, they will be directed to connect with the app support staff, who are available as avatars in the app 24/7.

**Table 1:** Outline of coach training.

	Training content	Training methods	Homework	Hours
1	Introductions; therapy versus peer support; types of meetings; CBI^a^ model; motivational interviewing; motivational interviewing roleplays	Group-based discussion; roleplays	Shadow two Innerworld meetings and send summaries to trainer	4
2	Review session 1 homework; intro to leading a meeting; overview of CBI tools	Didactics; live demonstrations; partner role-plays; group-based Q&A	Shadow two meetings; practice skills at home	4
3	Review session 2 homework; how to lead a meeting; how to teach the tools; continue to practice CBI tools	Live demonstrations; partner role-plays; group based Q&A	Shadow two meetings; practice skills with partner	4
4	Review session 3 homework; how to deal with trolls or minors; moderation tools; crisis situations; how to administer surveys	Live demonstrations; group-based Q&A; partner role-plays	Shadow two meetings; review crisis protocol	4
5	Review session 4 homework; practice roleplays and check-ins; discuss next steps to facilitating meetings	Group-based Q&A; group role-plays	Set up meeting for facilitation evaluation	4

^a^CBI: cognitive behavioral immersion.

**Table 2:** Outline of session content.

Session	Topic	Homework
1	Introduction to CBI^a^ model: icebreakers, practice with CBI conceptualization	Mood tracking
2	Behavioral activation and mood tracking: psychoeducation on mood tracking, behavioral activation, SMART^b^ goals	SMART goals
3	Identifying automatic thoughts: psychoeducation on automatic thoughts, cognitive distortions, introduction to thought record	Three-column thought record
4	Reframing automatic thoughts: catching distortions and reframing beliefs, individual practice with thought record and review as a group	Four-column thought record
5	Identifying and reframing core beliefs: psychoeducation on core beliefs, continued practice with thought record	Four-column thought record
6	Assertion training: psychoeducation on assertiveness curve tool and group role-plays to practice assertion skills	Assertion practice
7	Performance and arousal: overview of Yerkes-Dodson curve and how to target optimal arousal	Yerkes-Dodson practice
8	Relapse prevention plan and next steps: completing relapse prevention plan, course feedback, discuss key takeaways	Relapse prevention

^a^CBI: cognitive behavioral immersion.

^b^SMART: Specific, Measurable, Achievable, Relevant, and Time-Bound.

### Safety Monitoring

We will take precautions to monitor the safety of the study participants. First, all coaches and study investigators at USC will undergo training on how to manage crisis situations. During each CBI session, coaches will also monitor any discussion participants share regarding their safety in relation to themselves and others. We will also establish automatic thresholds on REDCap for PHQ-9 and 7-item Generalized Anxiety Disorder (GAD-7) assessments completed before, during, and after baseline. If a participant meets the criteria for suicidality (a 2 or above on the PHQ-9 suicide item), crisis resources will be automatically displayed and participants will be prompted to complete the Columbia-Suicide Severity Rating Scale (C-SSRS) Self-Report [33]. High-risk participants (scoring 4 or above on the C-SSRS Self Report) will be contacted by the research team via email to schedule a Zoom call for further assessment using the C-SSRS interview. Participants scoring a 4 or above on the C-SSRS interview will be excluded from the study due to sufficiently elevated suicide risk and will be provided with crisis and mental health resources. If imminent risk is present (C-SSRS interview score of 5), a lead study investigator (IDE) will evaluate the situation and contact local emergency services if deemed necessary. Participants with significantly worsening symptoms (a 20% increase on PHQ-9 or GAD-7 and a score of 15 or above) will be contacted by USC study personnel via email for a brief check-in and provided with mental health referrals. A copy of these referrals will also be publicly posted in the Innerworld app at all times.

All adverse incidents will be documented and immediately reported to lead study investigators, who will then follow them to a satisfactory resolution. They will distinguish serious adverse events from adverse events not judged to be serious using the guidance and definitions provided by the National Institutes of Health notices. All serious adverse events will be reported to the USC Institutional Review Board and the study’s independent Data and Safety Monitoring Board.

### Measures

#### Primary Outcome Measures

PHQ-9 will be used to assess depressive symptoms. The PHQ-9 [32] is a 9-item self-report measure with strong psychometric properties that mirrors the nine criteria for major depressive disorder in the Diagnostic and Statistical Manual, 4th edition. Scores range from 0 to 27, with higher scores indicating great severity.

The GAD-7 [34] will be used to assess participants’ generalized anxiety symptoms. The GAD-7 is a psychometrically strong 7-item self-report measure based on the diagnostic criteria for generalized anxiety disorder from the *Diagnostic and Statistical Manual of Mental Disorders* (Fourth Edition). Scores range from 0 to 21, with higher scores indicating greater severity.

#### Exploratory Measures

#### Outcomes

The World Health Organization Quality of Life-Brief [35] is a 26-item self-report measure of the quality of life within the domains of physical health, psychological health, social relationships, and environment. During the follow-up period, we will use only the first item to assess overall quality of life. Scores are transformed on a scale from 0 to 100, with higher scores indicating a higher quality of life. This measure has strong psychometric properties.

#### Prognostic Indices

The Competencies of Cognitive Therapy Scale is a 29-item self-report measure of three areas of cognitive behavioral skill competency: behavioral activation, automatic thoughts, and core beliefs [10]. Items are measured on 7-point scales, with higher scores indicating greater skill use. This measure has demonstrated strong psychometric properties.

The Group Session Rating Scale [36] is a 4-item measure that assesses group alliance and the quality of helper skills. Total scores range from 4 to 40 with higher scores indicating greater group alliance. This measure has demonstrated strong psychometric properties.

The Innerworld app will measure program engagement using passive user platform engagement (eg, sessions attended, minutes logged in, and type of device).

#### Prescriptive Indices

The Demographics Questionnaire is a basic demographic information survey that includes age, sex, gender identity, sexual orientation, race, ethnicity, educational attainment, employment or occupation, technology experience, past therapy experience, and prior diagnosis, among other variables.

The Immersion Questionnaire, adapted from a 3-item measure of social presence from Bailenson et al [37] and a 5-item measure of environmental presence from Fox et al [38], will be used to assess the degree to which the participant feels immersed in the metaverse.

#### Mechanisms

The Cognitive Change Immediate Scale [11] is a 5-item measure designed to assess participants’ experience of cognitive change and use of cognitive skills during sessions. Items are measured on a 7-point Likert scale, where lower scores indicate less agreement. This measure has demonstrated acceptable psychometric properties.

The Online Social Support Scale [39] is a 40-item scale with strong psychometric properties that assesses the level of social support participants receive from web-based sessions. We plan to use a 30-item version of the scale, measuring constructs of esteem or emotional support, social companionship, and informational support. Each item is scored from 1 to 5, with higher scores indicating a greater sense of social support.

### Storage and Timing of Assessments

All assessments will be managed by a research team at USC and securely collected using REDCap. All participants regardless of condition will receive the same set of web-based surveys at the same time points apart from the two session-related assessments (Group Session Rating Scale and Competencies of Cognitive Therapy Scale). These two session assessments will not be given to the DAC participants because they do not complete sessions during their 8-week condition. In the case that a participant does not complete a survey, study personnel will send up to three reminder emails over the course of 1 week post survey invitation to complete the uncompleted survey. See Table 3 for the assessment schedule.

**Table 3:** Assessment schedule.

Measures		Prescreen	Intake	Week 1	Week 2	Week 3	Week 4	Week 5	Week 6	Week 7	Week 8	Post	Follow up
**Screen**													
	Prescreen	✓											
**Outcome**													
	PHQ-9^a^	✓	✓	✓	✓	✓	✓	✓	✓	✓	✓	✓	✓
	GAD-7^b^		✓	✓	✓	✓	✓	✓	✓	✓	✓	✓	✓
	WHO^c^ Quality of Life-Brief		✓									✓	✓^d^
**Prognostic**													
	Competencies of Cognitive Therapy Scale		✓									✓	
	Group Session Rating Scale			✓	✓	✓	✓	✓	✓	✓	✓		
	Program engagement^e^	✓	✓	✓	✓	✓	✓	✓	✓	✓	✓	✓	✓
**Prescriptive**													
	Demographics Questionnaire		✓										✓^f^
	Immersion Questionnaire						✓					✓	
**Mechanism**													
	Cognitive Change Immediate Scale			✓	✓	✓	✓	✓	✓	✓	✓		
	Online Social Support Scale		✓				✓					✓	

^a^PHQ-9: 9-item Patient Health Questionnaire.

^b^GAD-7: 7-item Generalized Anxiety Disorder.

^c^WHO: World Health Organization.

^d^Only the first item from this measure is administered during the follow-up period.

^e^Continuous passive data collection.

^f^Participants will only be asked questions pertaining to changes in psychiatric medication or other psychological treatment during the follow-up period.

### Statistical Analysis

#### Descriptive Statistics

Data analysis will primarily use R statistical software (version 4.4.2; R Foundation for Statistical Computing) supplemented with other programs as needed. Theory-driven procedures (ie, inferential statistics) will use 2-tailed tests, α<.05, and power >80%. Descriptive statistics, including histograms, will be computed for each study variable, along with 95% bootstrap CIs. Data transformations will be made as needed. Depending on the distributional properties of variables, it may be necessary to use nonparametric statistics or generalized models with a link function to accommodate nonnormal distributions.

#### Primary Analysis

The main analytic approach will be a longitudinal random coefficients slope-as-outcome hierarchical linear model (HLM) conducted on the intent-to-treat sample. HLM is required for exploring the longitudinal nature of the data because repeated measurement of participants violates ANOVA’s assumption of independent errors. To control for correlated errors within participants, the model estimates an individual growth curve for each participant [40]. Advantages of this method over classical approaches include (1) repeated measurements increase statistical power, (2) shape of change over time is described, (3) missing values and unequal time intervals between measures for different participants can be handled, and (4) psychometric problems with change scores are avoided.

This study requires a 2-level HLM to represent multiple time points for each individual. Level 1, within participant, represents each participant’s time-varying symptoms as an individual linear trajectory. Level 2 uses individual fixed characteristics (eg, condition arm) to predict individual growth parameters (ie, participant’s intercept and slope). In this regression framework, we can then add covariates to ask more nuanced questions, observe interaction effects, and increase power by reducing error variance. We expect to estimate distinct linear slopes during the 8-week period (from baseline to postcondition) and the 6-month follow-up period (from postcondition to 6-month follow-up). Therefore, we plan to specify time in a piecewise manner to allow for separate slope estimates for the active condition and follow-up periods within the same regression model. This allows us to assess acute and long-term condition effects across the full duration of the trial.

Under the HLM framework, we will examine whether CBI-VR produces better clinical and functional outcomes compared to the CBI-FS and DAC conditions in terms of differences in trajectories (ie, slopes) and outcomes at each timepoint (ie, least-squares means and pairwise contrasts). Separate regression models will be used for each of the outcome variables (depression, anxiety, and quality of life). To examine differences in slopes, the primary effect of interest in the HLM model will be the Condition (CBI-VR, CBI-FS, and DAC) × Time (active condition and follow up) interaction effect. Using a piecewise specification of time, the models will yield estimates for the Condition × Time interaction effect during the acute condition and follow-up periods, corresponding to detecting acute and long-term differences in condition response (ie, slopes). We will also examine absolute change in outcome variables and differences in outcome variables by the condition at each timepoint through estimation of marginal or least-squares means and pairwise contrasts at each timepoint. This analysis will inform when outcomes start to differ during the active condition and the subsequent follow-up.

#### Exploratory Analysis

To explore baseline indices that predict general program response, we will enter proposed prognostic variables as predictors (ie, covariates) in HLM models using a stepwise approach.

To explore baseline indices that predict differential condition response, hypothesized prescriptive variables will be entered as potential moderators of program response in the HLM models. Specifically, separate Prescriptive Variable × Time interaction effects will be assessed in each HLM model. Again, the piecewise specification of time will allow for the prescriptive variables to be tested and identified during the active condition and follow-up periods.

To identify mechanisms of change of clinical and functional change across conditions, we will test for mediation of proposed mechanisms in the longitudinal framework by specifying hypothesized mediators as time-varying random effects. Estimation and inference for this time-varying mediation model will allow mediation effects to vary as a function of time. This method is suitable for detecting processes by which the intervention affects outcomes, as proposed mediators will be collected at multiple timepoints throughout the trial.

#### Sample Size

A longitudinal power analysis was conducted using the “powerlmm” package in R Statistical Software with α<.05, 2-tailed, and power >80%. Power analyses were focused on the ability to detect a medium effect size (Cohen *d*=0.50) at postcondition in the context of a 2-level random coefficients HLM. Given that the primary contrast of interest would be that between CBI and the other study conditions, calculations were focused on detecting significant differences between two active conditions. Where study parameters in this 2-level model were not yet known, we used standard values and varied parameters to approximate an effective sample size. We specified eight equally spaced timepoints, the amount of baseline variance at the subject level at 0.50, and allowed the ratio of the random slope variance to the within-subject variance to vary from 0.02 to 0.05 in increments of 0.01. Based on typical rates of drop out in digital CBT treatment, the rate of drop out was set at 25%. With anticipated missing data, the sample size estimate in each study arm varied from 65 to 138, yielding an average of 102. It is also recommended that power calculations should be based on the minimum detectable effect size or smallest difference between conditions that one would be willing to fail to detect [41]. We assessed this using the “PowerUpR” package (v1.1.0) [42] in R Statistical Software. Using the parameters specified above, we estimated a minimum detectable effect size of 0.26 (95% CI 0.08-0.44) with 102 in each study arm and a minimum detectable effect size of 0.22 (95% CI 0.07-0.37) with 138 in each study arm. With findings from Ezawa et al [29], we anticipate differences between conditions that will be considerably larger. Thus, a total sample size of 306 (n=102 in each study arm) will be effective for detecting meaningful differences in outcomes between CBI and the other conditions.

## Results

This study was funded in September 2023. Recruitment of study participants began in February 2024. As of January 2025, 306 participants have been enrolled. Data collection is expected to be completed by September 2025. Data have not yet been analyzed. We plan to submit initial results for publication in a peer-reviewed scientific journal and share them with ClinicalTrials.gov in the winter of 2025.

## Discussion

### Overview

This study will be the first randomized controlled trial to test the efficacy of CBI. While the cognitive behavioral skills being taught and the therapeutic framework of peer support are each supported empirically, this study investigates an intervention that integrates these components in a novel environment (the metaverse) and compares its efficacy to a minimal treatment control condition. This study will also examine the relative efficacy of accessing the coach-led cognitive behavioral skills training program via immersive virtual reality technology versus less immersive flat-screen devices. We anticipate that participants randomized to both CBI conditions will experience improvements in depression symptoms, anxiety symptoms, and quality of life. We also predict that participants who access the program through virtual reality will experience more positive outcomes than those who access the program through flat-screen devices. Furthermore, we will explore a variety of prognostic variables (ie, cognitive behavioral skills, group alliance, and program engagement), prescriptive variables (ie, demographics and immersion), and mechanisms (ie, cognitive change and social support) of program response.

The findings from this study will test the efficacy of this intervention relative to its absence and determine if and how the access platform affects efficacy. This study will also help to determine if there is a benefit to the increased cost of disseminating CBI through virtual reality headsets versus through devices that many people may already own. If deemed efficacious, this intervention can be promoted to scale and subsequently increase access to effective mental health interventions.

### Potential Challenges

We anticipate some potential challenges. The primary challenge we foresee is a high dropout rate given an elevated dropout rate among digital interventions in prior research [43]. To help reduce the risk of drop out, we have designed a comprehensive onboarding process to get study participants comfortable with the study procedures and address any anticipated technical barriers to study completion. We did incorporate an expected dropout rate of 25% into our power analysis to avoid low statistical power in the event of sizeable attrition rates. We will monitor participant engagement with the sessions and assessments, as well as send electronic reminders of intervention sessions and study assessments. If a participant misses any session or assessment, we will send follow-up reminders and inquire about barriers to completing the study. If any barriers are noted, we will work with the participant to address them. Given that this intervention is delivered on a web-based platform by nonprofessional coaches, there exists the chance that technical or personal issues related to Innerworld, or its coaches, may interfere with the delivery of the intervention to participants. To mitigate this risk, Innerworld provides 24/7 access to live coaches (who are trained on how to detect if a person is in crisis and provide the proper resources) and technical support to address any unexpected obstacles. Finally, we were unable to implement blinding due to differences between interventions (such as the device used to access the intervention) that would be obvious to participants, as well as coaches. This could introduce bias in how participants or coaches behave or perceive their symptoms and the intervention.

### Conclusions

If we find a significant difference in favor of CBI versus the DAC condition, this will provide evidence that CBI is efficacious for individuals with depression. If we find a significant difference in favor of CBI accessed via virtual reality headsets over the less immersive CBI accessed via flat-screen devices, this may suggest that the immersive quality of CBI strengthens the program. If we find no such difference, this will suggest that CBI may be effectively implemented with either virtual reality or flat-screen devices (ie, computer or handheld mobile devices), significantly reducing the cost of access since most people have a flat-screen device. This study will produce valuable information on whether CBI can effectively serve as a scalable and accessible mental health intervention among a growing, diverse population of help-seekers. In addition, knowledge of the differential benefit (or lack thereof) of immersive technology will help researchers develop other virtual reality and metaverse interventions, as well as funding agencies in determining whether virtual reality equipment is worthwhile or necessary in clinical studies.

## Appendix

Multimedia Appendix 1 CONSORT-eHEALTH checklist (V 1.6.1).

Multimedia Appendix 2 SPIRIT Checklist.

## Abbreviations

CBI: cognitive behavioral immersion
CBI-FS: cognitive behavioral immersion accessed via a flat-screen device
CBI-VR: cognitive behavioral immersion accessed via a virtual reality
CBT: cognitive behavioral therapy
CONSORT E-HEALTH: Consolidated Standards of Reporting Trials of Electronic and Mobile Health Applications and Online Telehealth
C-SSRS: Columbia-Suicide Severity Rating Scale
DAC: delayed access control
GAD-7: 7-item Generalized Anxiety Disorder
HLM: hierarchical linear modeling
PHQ-9: 9-item Patient Health Questionnaire
REDCap: Research Electronic Data Capture
SPIRIT: Standard Protocol Items: Recommendations for Interventional Trials
USC: University of Southern California

## Data Availability

Data sharing is not applicable to this article as no datasets have yet been analyzed during this study.
